# Use of 3-Deoxy-D-arabino-heptulosonic acid 7-phosphate Synthase (DAHP Synthase) to Enhance the Heterologous Biosynthesis of Diosmetin and Chrysoeriol in an Engineered Strain of *Streptomyces albidoflavus*

**DOI:** 10.3390/ijms25052776

**Published:** 2024-02-28

**Authors:** Álvaro Pérez-Valero, Juan Serna-Diestro, Claudio J. Villar, Felipe Lombó

**Affiliations:** 1 Research Group BIONUC (Biotechnology of Nutraceuticals and Bioactive Compounds), Departamento de Biología Funcional, Área de Microbiología, Universidad de Oviedo, 33006 Oviedo, Spain; apv.moratalla@gmail.com (Á.P.-V.); uo264212@uniovi.es (J.S.-D.); cjvg@uniovi.es (C.J.V.); 2 IUOPA (Instituto Universitario de Oncología del Principado de Asturias), 33006 Oviedo, Spain; 3 ISPA (Instituto de Investigación Sanitaria del Principado de Asturias), 33011 Oviedo, Spain

**Keywords:** flavonoid, biosynthesis, *O*-methyltransferase, regiospecificity, metabolic engineering

## Abstract

Flavonoids are a large family of polyphenolic compounds with important agro-industrial, nutraceutical, and pharmaceutical applications. Among the structural diversity found in the flavonoid family, methylated flavonoids show interesting characteristics such as greater stability and improved oral bioavailability. This work is focused on the reconstruction of the entire biosynthetic pathway of the methylated flavones diosmetin and chrysoeriol in *Streptomyces albidoflavus*. A total of eight different genes (TAL, 4CL, CHS, CHI, FNS1, F3′H/CPR, 3′-OMT, 4′-OMT) are necessary for the heterologous biosynthesis of these two flavonoids, and all of them have been integrated along the chromosome of the bacterial host. The biosynthesis of diosmetin and chrysoeriol has been achieved, reaching titers of 2.44 mg/L and 2.34 mg/L, respectively. Furthermore, an additional compound, putatively identified as luteolin 3′,4′-dimethyl ether, was produced in both diosmetin and chrysoeriol-producing strains. With the purpose of increasing flavonoid titers, a 3-Deoxy-D-arabino-heptulosonic acid 7-phosphate synthase (DAHP synthase) from an antibiotic biosynthetic gene cluster (BGC) from *Amycolatopsis balhimycina* was heterologously expressed in *S. albidoflavus*, enhancing diosmetin and chrysoeriol production titers of 4.03 mg/L and 3.13 mg/L, which is an increase of 65% and 34%, respectively. To the best of our knowledge, this is the first report on the de novo biosynthesis of diosmetin and chrysoeriol in a heterologous host.

## 1. Introduction

Flavonoids are a large class of polyphenols, representing around 9000 compounds that are widely distributed in plants [1,2,3]. These bioactive compounds are important in the nutraceutical, pharmaceutical, cosmetic, and agro-industrial fields due to the vast variety of properties they display [4,5,6], such as antitumor [7,8,9], antimicrobial, antiangiogenic [8,9], antioxidant, and neuroprotective compounds, among other bioactivities [10]. Flavonoids undergo diverse enzymatic modifications in their core structure, such as hydroxylations, glycosylations, or methylations, and in particular, methylated flavonoids possess interesting properties, such as major stability, improved oral bioavailability, enhanced membrane transport, and better intestinal absorption [11]. In the case of the methylated flavone diosmetin, which is widely present (as a glucosylated derivative) in several *Citrus* fruits, it has been reported to have antioxidant, phlebotonic, oestrogenic, antimicrobial, and anti-inflammatory activities [12]. As an antimicrobial, it should be noted that in combination with erythromycin, diosmetin shows a synergistic effect against methicillin-resistant *Staphylococcus aureus* (MRSA) [13]. On the other hand, chrysoeriol, another luteolin methylated derivative, has been found in several plants, such as *Reseda luteola*, *Melientha suavis*, and *Cardiospermum halicacabum* L. This methylated flavonoid is of interest due to its potential as an anti-hyperlipidemic, antitumor, antioxidant, antimicrobial, antifungal, and neuroprotective agent, among other bioactivities [14].

Depending on their structural differences, flavonoids are classified into seven subclasses: flavanones, flavones, isoflavones, flavonols, anthocyanidins, flavanols, and chalcones [15]. For the heterologous biosynthesis of flavonoids in bacteria, several enzymatic steps must take place, starting with the conversion of L-tyrosine in coumaric acid by the tyrosine ammonia lyase (TAL). Then, coumaric acid should be activated with the molecule coenzyme-A by the action of 4-coumaroyl-CoA ligase (4CL), giving rise to 4-coumaroyl-CoA, which would be then condensed with three molecules of malonyl-CoA through chalcone synthase (CHS), generating naringenin chalcone, the basic carbon skeleton for all flavonoids known in nature [16,17,18,19]. Finally, the heterocycle closure in naringenin chalcone is carried out by a chalcone isomerase (CHI), generating the universal flavanone precursor, naringenin. For the biosynthesis of diosmetin and chrysoeriol, three extra enzymatic steps are necessary. Naringenin should be converted to apigenin by a flavone synthase. In the bacterial host *S. albidoflavus*, a class I flavone synthase (FNS1) is necessary, lacking the need for a cytochrome P450 membrane-bound monooxygenase, which would be difficult to express in bacteria [20]. Apigenin should then be converted to luteolin by a flavone 3′ hydroxylase. In this work, a flavone 3′ hydroxylase coupled with a soluble cytochrome P450 reductase (F3′H-CPR) has been used, providing the reducing power to the flavone 3′ hydroxylase [21]. Finally, a 4′-*O*-methyltransferase (4′OMT) is necessary to reach the heterologous biosynthesis of diosmetin, and a 3′-*O*-methyltransferase (3′OMT) is required for the heterologous production of chrysoeriol.

The biosynthesis of methylated flavonoids has been achieved using different microbial cell factories, such as *Escherichia coli* [22] and *Saccharomyces cerevisiae* [23]. Additionally, in recent work by our research group, the biosynthesis of this type of flavonoid has been achieved in the Gram-positive bacterium *S. albidoflavus* [24]. However, a major bottleneck in the heterologous biosynthesis of flavonoids in bacteria using synthetic biology tools is the low production titters. Several strategies have been applied to increase the intracellular pools of flavonoid precursors, such as the redirection of the carbon source for the bacteria towards the biosynthesis of malonyl-CoA [25], a key precursor in the flavonoid pathway.

So far, the biosynthesis of diosmetin and chrysoeriol had never been carried out in a heterologous host. In this work, we present the production of both methylated flavones after dissecting their biosynthetic pathways into three modules, leveraging different integration sites of the bacteriophages ϕC31 [26] and ϕBT1 [27], in addition to the integration site of the pSAM2 plasmid [28] in the chromosome of *S. albidoflavus*, enabling the stable incorporation of exogenous DNA into the bacterial chromosome (across three different sections, corresponding to three different steps during flavonoid biosynthesis) and avoiding the necessity for antibiotic selection to maintain plasmids.

As a further step towards the enhancement of the final production titers of these two methylated flavones in *S. albidoflavus*, the gene encoding a DAHP synthase from the actinomycete *Amycolatopsis balhimycina* [29] has been added together with the diosmetin and chrysoeriol biosynthetic pathways. This enzyme, DAHP, carries out the condensation of phosphoenolpyruvate (PEP) and erythrose 4-phosphate (E4P) [30], the first enzymatic reaction in the shikimate pathway towards the biosynthesis of aromatic amino acids, such as the flavonoid precursor L-tyrosine.

The current industrial production of diosmetin is based on semi-synthesis approaches from its glycosylated form, diosmin. This semi-synthesis makes use of concentrated sulfuric acid and several crystallization steps, which lower the final efficiency of the whole process. Other industrial alternatives include the use of hesperidin (a flavanone from orange peels), which is oxidized under hot alcoholic sodium acetate (with its sugar moiety removed chemically) to generate diosmetin [31,32]. In this work, the need for flavonoid precursors (hesperidin, diosmin, etc.) is not necessary for obtaining the final bioactive compounds, as these are generated de novo, using common metabolic intermediates available in the bacterial cytoplasm.

## 2. Results

### 2.1. Heterologous Biosynthesis of Diosmetin

**Figure 1:**
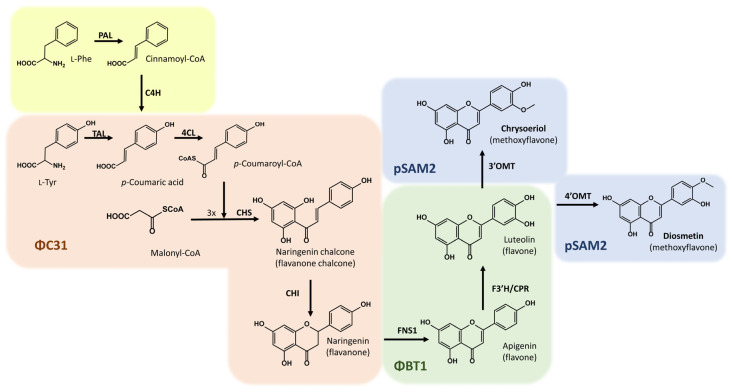
Biosynthetic pathway for the biosynthesis of diosmetin and chrysoeriol. The yellow section indicates the canonical first steps in plants. Tyrosine ammonia-lyase (TAL); 4-Coumaroyl-CoA ligase (4CL); Chalcone synthase (CHS); Chalcone isomerase (CHI); Flavone synthase (FNS1); Flavonoid 3′ hydroxylase/Cytochrome P450 reductase chimera (F3′H/CPR); 4′-*O*-methyltransferase (4′OMT); 3′-*O*-methyltransferase (3′OMT). In light orange, the part of the pathway integrated into the ϕC31 *attb* site of the chromosome is shown; in light green, the part of the pathway integrated into the ϕBT1 *attb* site of the chromosome is shown; in light blue, the final part of the pathway for the heterologous biosynthesis of diosmetin or chrysoeriol is shown, with both cases integrated into the pSAM2 site of the *S. albidoflavus* chromosome. The yellow section indicates the naturally occurring pathway in plants, using L-phenylalanine instead of L-tyrosine.

For the biosynthesis of diosmetin in bacteria, the action of seven enzymes (TAL, 4CL, CHS, CHI, FNS1, F3′H-CPR, and 4′OMT) is required (in contrast to the eight necessary genes in plants). In this work, this whole biosynthetic pathway was divided into three modules (Figure 1), and the necessary coding genes were distributed along the chromosome of the strain *S. albidoflavus* UO-FLAV-004 [24], taking advantage of the different prophage integration sites in this species. All the genes were codon-optimized for *S. albidoflavus*. TAL, 4CL, CHS, and CHI genes were integrated into the ϕC31 *attb* site [24], FNS1 [24] and F3′H-CPR [21] genes were integrated into the ϕBT1 *attb* site, and the 4′OMT gene was integrated into the pSAM2 site of the chromosome.

The selected 4′OMT is a hypothetical class I SAM-dependent *O*-methyltransferase (M444_29925) from *Streptomyces* sp. Mg1 (see Section 4). This gene was assembled under the control of the SP25 promoter, which shows good transcriptional activity, being higher than the widely used PermE* promoter [33]. The gene *M444_29925* was selected after a BLASTP analysis against the *O*-methyltransferase GerMIII, which shows a relative in vitro conversion rate of luteolin to diosmetin of 67% [34]. The differences between *GerMIII* and *M444_29925* at the protein level lie in the amino acids 81 (A-P) and 377 (T-I).

**Figure 2:**
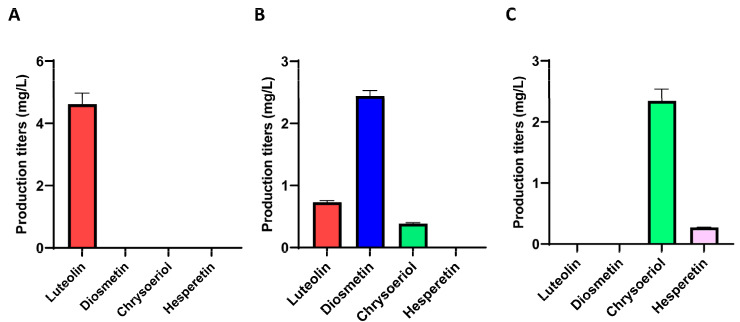
Production titers of different flavonoids in different strains derived from *S. albidoflavus* UO-FLAV-004. (**A**) *S. albidoflavus* UO-FLAV-004-LUT; (**B**) *S. albidoflavus* UO-FLAV-004-DIO; (**C**) *S. albidoflavus* UO-FLAV-004-CHR.

After the corresponding fermentation experiments, the control strain *S. albidoflavus* UO-FLAV-004-LUT, which harbors the genes TAL, 4CL, CHS, CHI, FNS1, and F3′H-CPR, as well as an empty plasmid integrated into the pSAM2 chromosomal site, was able to produce the unmethylated precursor of diosmetin and luteolin, reaching titers of 4.61 mg/L (Figure 2A). The diosmetin-producing strain *S. albidoflavus* UO-FLAV-004-DIO, which additionally containins the gene coding for the M444_29925 4′-*O*-methyltransferase integrated into the pSAM2 chromosomal site, generated 2.44 mg/L of diosmetin and 0.73 mg/L of luteolin. Surprisingly, a small peak of chrysoeriol was also detected in the chromatogram, indicating the capability of this strain to produce the luteolin 3′-*O*-methyl ether (chrysoeriol) at titers of 0.38 mg/L as well (Figure 2B).

### 2.2. Heterologous Biosynthesis of Chrysoeriol

The heterologous biosynthesis of chrysoeriol was carried out using the same distribution of genes as in the previous case. The same enzymatic activities required for the heterologous biosynthesis of diosmetin were necessary to produce the common precursor, luteolin, but a different *O*-methyltransferase was needed to introduce the methyl moiety at position 3′ instead of position 4′ of the ring B of luteolin (Figure 1). The 3′-*O*-methyltransferase used in the biosynthesis of chrysoeriol was a CCoAOMT-like enzyme from *Arabidopsis thaliana* (At4g26220), whose gene was optimized for *S. albidoflavus* (Table 1). This enzyme possessed a greater preference for introducing a methyl group in the *para* position (4′) in flavanones and dihydroflavonols, whereas flavones and flavonols were methylated in the meta position (3′) [35]. Using this enzyme, the biosynthesis of chrysoeriol in the strain *S. albidoflavus* UO-FLAV-004-CHR was achieved, reaching titers of 2.34 mg/L, with no remaining luteolin detected. Also, a small peak corresponding to hesperetin was detected in the chromatogram of this culture extract, and its production reached 0.27 mg/L (Figure 2C). The presence of this compound in this extract will be addressed in the Section 3. The strain used as control for this fermentation was *S. albidoflavus* UO-FLAV-004-LUT, as in the previous case.

### 2.3. Identification of Putative Luteolin 3′,4′-Dimethyl Ether in Both Diosmetin- and Chrysoeriol-Producing Strains

**Figure 3:**
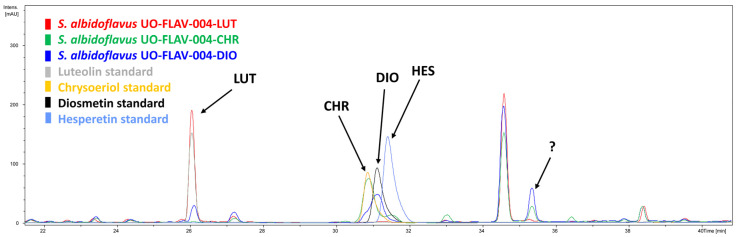
HPLC-DAD chromatograms of the strains *S. albidoflavus* UO-FLAV-004-LUT (red), *S. albidoflavus* UO-FLAV-004-CHR (green), *S. albidoflavus* UO-FLAV-004-DIO (blue). The four commercial standards are also indicated. Luteolin (LUT); Chrysoeriol (CHR); Diosmetin (DIO); Hesperetin (HES); Unknown differential peak (?).

After analyzing the chromatograms of the two different producing strains, an extra differential peak was also detected at late retention times (35.4 min), with a higher intensity in the case of the strain *S. albidoflavus* UO-FLAV-004-DIO (Figure 3). A late retention time indicates less polarity in the used HPLC program (see Section 4), which suggested that this peak could correspond to di-methylated luteolin, a less polar compound than a single methylated luteolin. The available positions in diosmetin for *O*-methylation were positions 5 and 7 of ring A and position 3′ of ring B, while for chrysoeriol, the available positions were positions 5 and 7 of ring A and position 4′ of ring B. Since standards for all possible combinations were not available, a feeding assay was performed to confirm or discard our first putative candidate, which putatively was considered luteolin 3′,4′-dimethyl ether.

**Figure 4:**
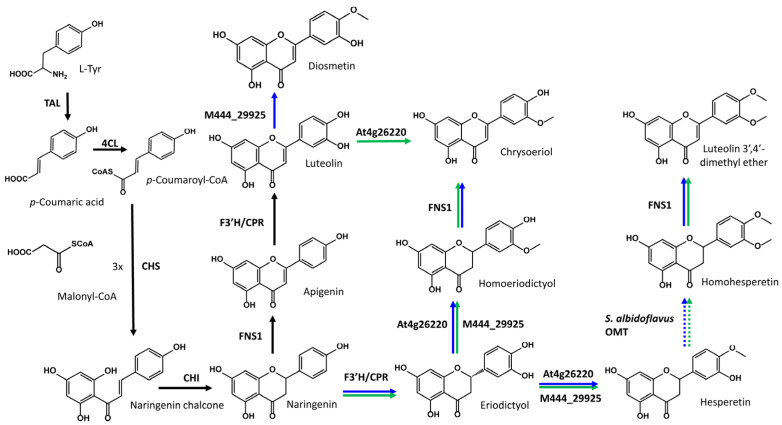
Proposed heterologous biosynthetic pathway for the production of diosmetin, chrysoeriol, and luteolin 3′,4′-dimethyl ether in *S. albidoflavus*. Black arrows connect the necessary reactions to reach the biosynthesis of luteolin. Blue arrows represent the reactions carried out in the strain *S. albidoflavus* UO-FLAV-004-DIO. Finally, green arrows represent the reactions carried out in the strain *S. albidoflavus* UO-FLAV-004-CHR. Dashed arrows indicate a reaction carried out by an endogenous enzyme of *S. albidoflavus*.

In the strains *S. albidoflavus* UO-FLAV-004-DIO and *S. albidoflavus* UO-FLAV-004-CHR, one of the enzymes in the heterologous biosynthetic pathway was the FNS1 flavone synthase from *Petroselinum crispum*, which has been described as being able to transform homoeriodictyol to chrysoeriol with high efficiency [36] (Figure 4). Taking this into account, since the strain *S. albidoflavus* UO-FLAV-004-CHR harbored the necessary genes for the biosynthesis of hesperetin (eriodictyol 4′-*O*-methyl ether), such as the *O*-methyltransferase At4g26220 [35] (Figure 4), hesperetin could be converted to homohesperetin (eriodictyol 3′,4′-dimethyl ether) by an endogenous *O*-methyltransferase activity of *S. albidoflavus* (see Section 3). Due to the structural similarity between homoeriodictyol (eriodictyol 3′-*O*-methyl ether) and homohesperetin (eriodictyol 3′,4′-*O*-methyl ether), we hypothesize that the FNS1 enzyme could be acting on the homohesperetin flavanone to produce the luteolin 3′,4′-dimethyl ether flavone (Figure 4).

With the aim of proving the presence of this extra activity in FNS1, a feeding was made with homohesperetin at a final concentration of 0.1 mM to the strain *S. albidoflavus* UO-FLAV-004-FNS1, previously developed by our research group [24], and to the strain *S. albidoflavus* UO-FLAV-004 as a control. Analysis of the HPLC-DAD chromatograms generated showed the presence of a derivative peak in the strain containing FNS1, which was co-eluting with the extra peaks observed in the diosmetin- and chrysoeriol-producing strains. This new peak was also showed the same UV absorption spectrum as the putative luteolin 3′,4′-dimethyl ether from the diosmetin- and chrysoeriol-producing strains (Figure S1). These results suggested that the strain *S. albidoflavus* UO-FLAV-004-CHR was able to produce luteolin 3′,4′-dimethyl ether through this pathway (Figure 4), and it indicated that homohesperetin could be a good substrate for the FNS1 enzyme.

On the other hand, the strain *S. albidoflavus* UO-FLAV-004-DIO, which also produced this putative luteolin di-methylated derivative, albeit at a slightly higher concentration, should not be able to produce it through the same pathway as *S. albidoflavus* UO-FLAV-004-CHR, since the gene *At4g26220* was not present in this strain. A feasible alternative to explain the production of this compound in this strain was the conversion of eriodictyol to hesperetin by the action of the enzyme 4′OMT of *Streptomyces* sp. Mg1, an activity that had not been reported so far. To check the substrate flexibility of this enzyme, a feeding experiment using eriodictyol was performed on the strain *S. albidoflavus* UO-FLAV-004-M444_29925, harboring only the 4′OMT of *Streptomyces* sp. Mg1 in the pSAM2 chromosomal integration site, along with the corresponding control strain *S. albidoflavus* UO-FLAV-004 harboring an empty plasmid in the same chromosomal *attb* site. The feeding results were analyzed by HPLC-DAD and showed a good conversion of eriodictyol to both hesperetin (eriodictyol 4′-*O*-methyl ether) and homoeriodictyol (eriodictyol 3′-*O*-methyl ether) in *S. albidoflavus* UO-FLAV-004-M444_29925. Additionally, a peak of homohesperetin was detected after the feeding (Figure S2). No eriodictyol derivative was observed in the control strain. This suggested that hesperetin was generated in the strain *S. albidoflavus* UO-FLAV-004-DIO by the enzyme M444_29925 and converted to homohesperetin by the putative endogenous *O*-methyltransferase activity of *S. albidoflavus*, finally being converted to luteolin 3′,4′-dimethyl ether by the action of FNS1, like in the case observed in the strain *S. albidoflavus* UO-FLAV-004-CHR (Figure 4).

### 2.4. Use of a DAHP Synthase to Increase the Production Titers of Diosmetin and Chrysoeriol through Precursor Titer Enhancement

**Figure 5:**
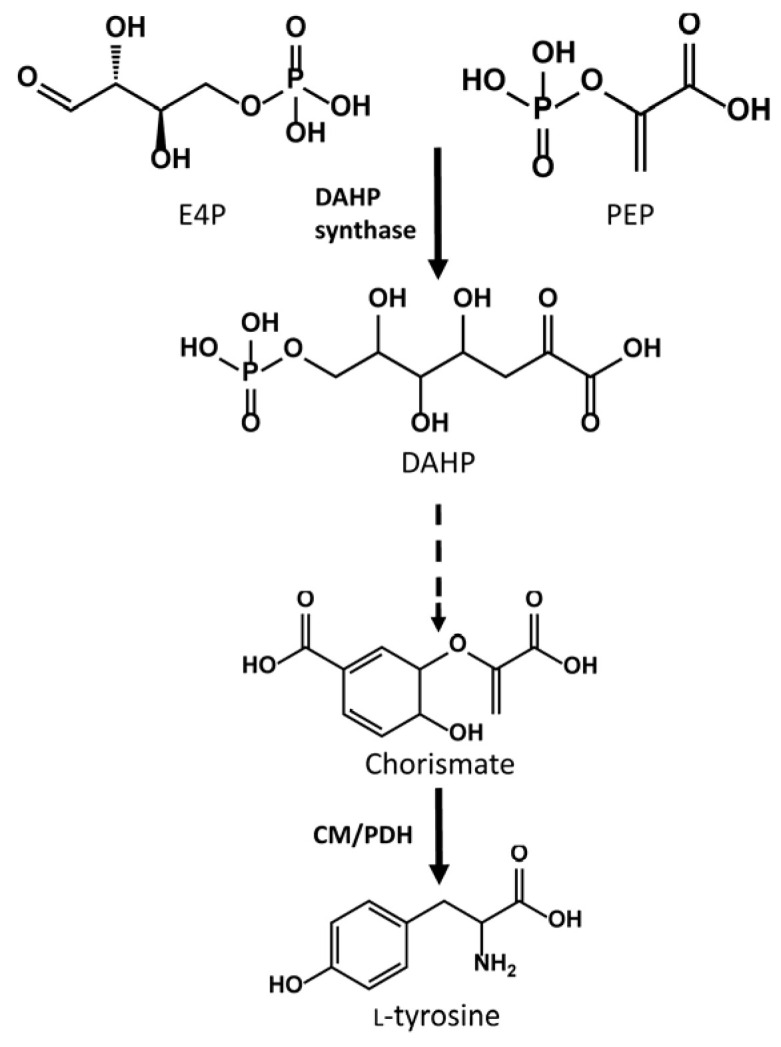
Abbreviated schema of the shikimate pathway for the generation of L-tyrosine. E4P: Erythrose 4-phosphate; PEP: phosphoenolpyruvate; DAHP: 3-Deoxy-D-arabino-heptulosonic acid 7-phosphate: CM/PDH: chorismate mutase/prephenate dehydrogenase.

DAHP synthase is the first enzyme of the shikimate pathway. This enzyme condenses D-erythrose 4-phosphate and phosphoenolpyruvate to produce DAHP, a key precursor in the biosynthesis of aromatic amino acids, such as L-tyrosine (Figure 5). L-tyrosine is the first precursor in the heterologous pathway for flavonoid biosynthesis in *S. albidoflavus*, making the overexpression of the DAHP synthase an interesting strategy to enhance the intracellular pools of L-tyrosine and, thus, the final flavonoid titers. This strategy was followed by Thykaer and colleagues to increase the titers of the vancomycin analogue balhimycin in the natural producing strain of *Amycolatopsis balhimycina*, resulting in improved specific productivities of balhimycin by introducing an extra copy of the *dahp* gene [29].

**Figure 6:**
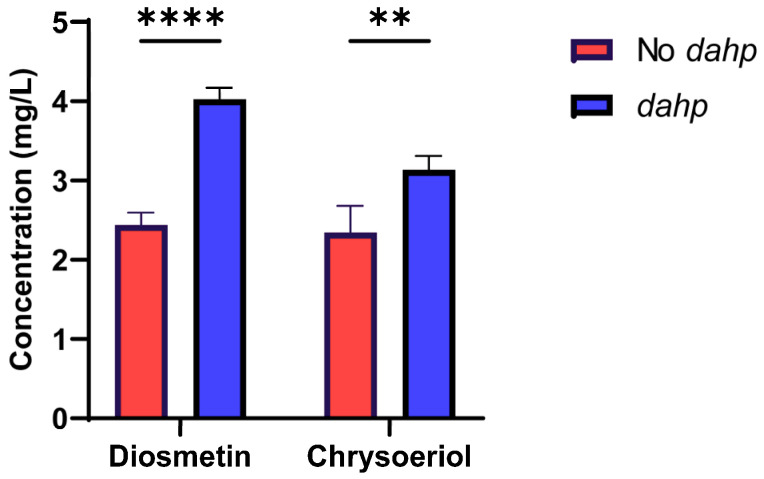
Effect of the DAHP synthase on the biosynthesis of diosmetin and chrysoeriol in the strains *S. albidoflavus* UO-FLAV-004-DIO-dahp and *S. albidoflavus* UO-FLAV-004-CHR-dahp compared to the strains *S. albidoflavus* UO-FLAV-004-DIO and *S. albidoflavus* UO-FLAV-004-CHR, respectively. Asterisks indicate statistically significant differences (** *p* <0.005; **** *p* <0.0001).

In this work, a codon optimization of the *dahp* gene of *Amycolatopsis balhimycina* was conducted for *S. albidoflavus*, and the gene was assembled under the control of the strong SP25 promoter. Then, this gene was brought together with the different *O*-methyltransferases involved in diosmetin and chrysoeriol biosynthesis (see Section 4) and integrated into the pSAM2 chromosomal site of *S. albidoflavus* UO-FLAV-004-LUT parental strain, giving rise to the final strains *S. albidoflavus* UO-FLAV-004-DIO-dahp and *S. albidoflavus* UO-FLAV-004-CHR-dahp, respectively. These new strains were cultivated at the same time as the strains *S. albidoflavus* UO-FLAV-004-DIO and *S. albidoflavus* UO-FLAV-004-CHR, used as controls, to check if the enzyme DAHP synthase was able to increase the titers of diosmetin and chrysoeriol. The resulting production titers were 4.03 mg/L of diosmetin for *S. albidoflavus* UO-FLAV-004-DIO-dahp and 3.13 mg/L of chrysoeriol for *S. albidoflavus* UO-FLAV-004-CHR-dahp, which represents a 1.65-fold and 1.34-fold increase, respectively (Figure 6). Also, as expected, the by-products chrysoeriol and hesperetin were present in the strains *S. albidoflavus* UO-FLAV-004-DIO and *S. albidoflavus* UO-FLAV-004-CHR, respectively, as well as the putative luteolin 3′,4′-dimethyl ether, all in proportionally increased quantities in the strains *S. albidoflavus* UO-FLAV-004-DIO-dahp and *S. albidoflavus* UO-FLAV-004-CHR-dahp (Figures S3 and S4).

## 3. Discussion

Previous studies conducted by our research group have revealed the potential of *S. albidoflavus* for the biosynthesis of methylated derivatives of flavonoids [24]. To our knowledge, this work describes, for the first time, the complete biosynthesis of the methylated flavonoids diosmetin and chrysoeriol in a heterologous host.

The enzyme M444_29925 shares 98% identity with the well-studied GermIII *O*-methyltransferase, which shows high regiospecificity for the 4′ position of flavones [34]. Along the biosynthetic pathway of diosmetin, the reactions can also occur in a different order. If the chimeric F3′H-CPR hydroxylase uses naringenin as a substrate before FNS1, the strain will first produce eriodictyol. A difference between flavones and flavanones is the spatial configuration of their chemical structures. Flavones present a planar structure [37], while flavanones present a chair conformational structure [38]. According to this, we hypothesize that the enzyme M444_29925 may introduce a methyl group in a different position in a flavanone structure, such as eriodictyol, instead of the 4′ position, as observed in flavones. With the aim of finding out if *O*-methylation in a different position is possible for this enzyme, a feeding experiment with eriodictyol was performed on a strain containing only the *O*-methyltransferase M444_29925, named *S. albidoflavus* UO-FLAV-004-M444_29925, and the strain *S. albidoflavus* UO-FLAV-004 as a control. As shown previously, we verified that this enzyme was able to methylate eriodictyol, at least, at positions 4′ and 3′, yielding hesperetin (eriodictyol 4′-*O*-methyl ether) and homoeriodictyol (eriodictyol 3′-*O*-methyl ether) (Figure 4). The generation of homoeriodictyol through this enzyme can explain the presence of chrysoeriol in the strain *S. albidoflavus* UO-FLAV-004-DIO due to the action of FNS1 (Figure 4) [36].

On the other hand, the presence of hesperetin in the chrysoeriol-producing strain is easy to explain since the CcoAOMT-like enzyme At4g26220 efficiently produces hesperetin from eriodictyol in vitro (Figure 4) [35]. At4g26220 also produces homoeriodictyol [35], but again, FNS1 converts it to chrysoeriol.

Finally, as described in the Section 2, homohesperetin was detected after the administration of eriodictyol to the strain *S. albidoflavus* UO-FLAV-004-M444_29925. Given that luteolin 3′,4′-dimethyl ether is a direct derivative of homohesperetin following enzymatic mediation by FNS1, and this proposed compound is discernible in both *S. albidoflavus* UO-FLAV-004-DIO and *S. albidoflavus* UO-FLAV-004-CHR strains, it is inferred that homohesperetin should be inherently present in both the diosmetin- and chrysoeriol-producing strains. This deduction is supported by the detection of the putative luteolin 3′,4′-dimethyl ether in both strains. Consequently, these results imply that homohesperetin is likely produced through an unknown endogenous activity of *S. albidoflavus* in the presence of hesperetin and/or homoeriodictyol.

Thus, if homohesperetin is found in the bacterial cytoplasm at any moment during the biosynthesis of diosmetin and chrysoeriol, it can be transformed into luteolin 3′,4′-dimethyl ether by the action of the FNS1. These results highlight the important role that substrate specificity of the selected enzymes plays in the heterologous biosynthesis of natural products, and it may be the case that these enzymes compete for different molecules found at different stages of a given pathway, generating different derivatives. However, this could also be advantageous since new unexpected products could be obtained, such as the putative luteolin 3′,4′-dimethyl ether, establishing new pathways to produce them.

Regarding the biosynthesis of diosmetin and chrysoeriol, attempts were made to increase production titers by using a DAHP synthase. The biosynthesis of aromatic amino acids is strictly regulated by feedback inhibition mechanisms, and DAHP synthases are normally feedback regulated by L-tyrosine, L-phenylalanine, or both [39]. The strategy of using a DAHP synthase to enhance the flavonoid titters has been carried out by different researchers. Koopman and colleagues achieved an increase in naringenin titters using different approaches in *Saccharomyces cerevisiae*, including the alleviation of feedback inhibition of yeast DAHP synthase by introducing a L-tyrosine insensitive ARO4 (*ARO4*^G226S^) allele in conjunction with the deletion of the other allele of the *dahp* gene [40]. In another experiment in *E. coli*, the overexpression of a feedback-resistant derivative of *dahp* (*aroG*^fbr^), together with the overexpression of chorismate mutase/prephenate dehydrogenase (*tyrA*^fbr^), led to an increase in naringenin [41,42], apigenin, and genkwanin titters [43].

Here, a DAHP synthase from the actinomycete *Amycolatopsis balhimycina* has been used, which has been previously proved as a useful metabolic engineering strategy to increase the biosynthesis of glycopeptide antibiotics by introducing an extra copy in its natural producer. In *A. balhimycina*, the gene encoding this DAHP synthase is found in a chromosomal region containing a BGC and is not involved in primary metabolism [29]. Other *dahp* genes have been identified in other BGCs [44,45], and in these cases, the *dahp* genes were like those encoding the plant type DAHP synthases, which were proven to be naturally resistant to feedback inhibition by aromatic amino acids [46,47]. Here, we have selected this gene to be assayed in the engineered strain *S. albidoflavus* UO-FLAV-004, as it lacks the negative feedback inhibition by the final shikimate pathway product (L-tyrosine). In this way, a significant increase in the biosynthesis of diosmetin (1.65-fold) and chrysoeriol (1.34-fold) has been achieved, proving the positive effect on flavonoid biosynthesis of this DAHP synthase gene from *Amycolatopsis balhimycina*, placed in this case under the regulation of a strong constitutive promoter.

This study serves as proof of the suitability of *S. albidoflavus* for the biosynthesis of methylated flavonoids and reveals the efficacy of using a gene coding for a DAHP synthase from a BGC of another actinomycete bacterium, which lacks feedback inhibition by aromatic amino acids (such as the primary flavonoid precursor L-tyrosine), therefore allowing the enhancement of flavonoid titers.

## 4. Materials and Methods

### 4.1. Bacterial Strains and Culture Conditions

All strains used in this study are listed in Table 1. *Escherichia coli* TOP10 (Invitrogen, Waltham, MA, USA) was used for routine subcloning. *E. coli* ET12567/pUZ8002 [48] was used for conjugation. The strain used in this study for the heterologous biosynthesis of diosmetin and chrysoeriol was *S. albidoflavus* UO-FLAV-004-NAR [24]. To achieve the heterologous biosynthesis of these two methylated flavones, their biosynthetic pathways were divided into three parts. The first part is the BGC for naringenin biosynthesis, containing the enzymes TAL, 4CL, CHS, and CHI. The genes coding for the enzymes of this BGC were already integrated into the ϕC31 *attb* site of the strain *S. albidoflavus* UO-FLAV-004-NAR. A second plasmid containing the genes coding for the enzymes FNS1 and the chimera F3′H-CPR was integrated into the ϕBT1 *attb* site, giving rise to the strain *S. albidoflavus* UO-FLAV-004-LUT, which is able to produce luteolin. Over this last strain, a third plasmid integration was performed into the pSAM2 site, involving the plasmids pSEVAUO-M31105-M444_29925 or the plasmid pSEVAUO-M31105-At4g26220, generating the strains *S. albidoflavus* UO-FLAV-004-DIO or *S. albidoflavus* UO-FLAV-004-CHR, respectively.

**Table 1:** Plasmids and strains used in this study.

	Description	Reference
**Plasmids**		
pSEVA181-At4g26220	Source of *At4g26220* (Level 0 MoClo)	This study
pSEVA181SP25	Source of *SP25* (Level 0 MoClo)	[21]
pSEVA181SP43	Source of *SP43* (Level 0 MoClo)	[21]
pSEVA181-M444_29925	Source of *M444_29925* (Level 0 MoClo)	This study
pSEVA181RiboJ-RBS	Source of *RiboJ-RBS* (Level 0 MoClo)	[21]
pIDTSMARTttsbib	Source of *ttsbib* (Level 0 MoClo)	[21]
pSEVAUO-M21102	Level 2 MoClo receptor	[21]
pSEVAUO-M31205	Level 2 MoClo receptor	[21]
pSEVAUO-M21206F3H-CPR	Level 1 MoClo harboring *F3′H-CPR*	[21]
PCR-Blunt II-TOPO-FNS1	Source of *FNS1* (Level 0 MoClo)	[24]
pSEVAUO-M21102-FNS1	Level 1 MoClo harboring *FNS1*	This study
pSEVAUO-M21503-FNS1/F3′H-CPR	Level 2 MoClo harboring *FNS1* and *F3′H-CPR*	This study
pSEVAUO-M31105-At4g26220	Level 1 MoClo plasmid harboring *At4g26220*	This study
pSEVAUO-M31105-M444_29925	Level 1 MoClo plasmid harboring *M444_29925*	This study
pSEVAUO-M31105	Level 1 MoClo receptor	[21]
pSEVAUO-M31205-dahp	Level 1 MoClo plasmid harboring *dahp*	This study
pSEVAUO-M31505	Level 2 MoClo receptor	[21]
pSEVAUO-M31505-At4g26220-dahp	Level 2 MoClo harboring *At4g26220* and *dahp*	This study
pSEVAUO-M31505-M444_29925-dahp	Level 2 MoClo harboring *M444_29925* and *dahp*	This study
**Strains**		
*E. coli* TOP10	Strain used for routine subcloning	Invitrogen (Waltham, MA, USA)
*E. coli* ET12567/pUZ8002	Strain used for conjugation	[48]
UO-FLAV-004	*S. albidoflavus* strain used in this work	[24]
UO-FLAV-004-NAR	UO-FLAV-004 harboring *TAL*, *4CL*, *CHS* and *CHI*	[24]
UO-FLAV-004-LUT	UO-FLAV-004 harboring *TAL*, *4CL*, *CHS*, *CHI*, *FNS1* and *F3′H-CPR*	This study
UO-FLAV-004-DIO	UO-FLAV-004 harboring *TAL*, *4CL*, *CHS*, *CHI*, *FNS1*, *F3′H-CPR* and *M444_29925*	This study
UO-FLAV-004-CHR	UO-FLAV-004 harboring *TAL*, *4CL*, *CHS*, *CHI*, *FNS1*, *F3′H-CPR* and *At4g26220*	This study
UO-FLAV-004-DIO-dahp	UO-FLAV-004 harboring *TAL*, *4CL*, *CHS*, *CHI*, *FNS1*, *F3′H-CPR*, *M444_29925* and *dahp*	This study
UO-FLAV-004-CHR-dahp	UO-FLAV-004 harboring *TAL*, *4CL*, *CHS*, *CHI*, *FNS1*, *F3′H-CPR*, *At4g26220* and *dahp*	This study
UO-FLAV-004-FNS1	UO-FLAV-004 harboring FNS1	[24]

Also, two more strains were generated over *S. albidoflavus* UO-FLAV-004-LUT. A MoClo level 2 plasmid was integrated into the pSAM2 chromosomal site containing the genes coding for the M444_29925 *O*-methyltransferase and the DAHP enzyme, generating the strain *S. albidoflavus* UO-FLAV-004-DIO-dahp. In the same manner, a plasmid containing the gene coding for the At4g26220 *O*-methyltransferase plus the DAHP enzyme was integrated in the same position, generating the strain *S. albidoflavus* UO-FLAV-004-CHR-dahp. In order to perform different feeding experiments, the strain *S. albidoflavus* UO-FLAV-004-M444_29925 was generated by transforming the strain *S. albidoflavus* UO-FLAV-004 with the plasmid pSEVAUO-M31105-M444_29925. Finally, the strain *S. albidoflavus* UO-FLAV-004-FNS1 [24] was also used for a feeding experiment.

*E. coli* strains were grown in tryptic soy broth (TSB, VWR, Barcelona, Spain) or on TSB agar plates, supplemented with the corresponding antibiotics (ampicillin 100 µg/mL, Sigma Aldrich, Madrid, Spain; apramycin 100 µg/mL, Thermo Fisher Scientific, Waltham, MA, USA); kanamycin 100 µg/mL (Alfa Aesar, Karlsruhe, Germany), chloramphenicol 25 µg/mL (AppliChem, Barcelona, Spain), nalidixic acid 50 µg/mL (Acros Organics, Geel, Belgium), and X-gal (AppliChem, Darmstadt, Germany) when blue-white selection was needed. *S. albidoflavus* was grown and sporulated at 30 °C in Bennett medium [49] supplemented with the corresponding antibiotics when necessary (thiostrepton 50 µg/mL, Cayman Chemical, Ann Arbor, MI, USA; hygromycin B 200 µg/mL, Enzo, Barcelona, Spain, or apramycin 50 µg/mL). MA medium (plus 10 mM MgCl2) was used for conjugation between *S. albidoflavus* and *E. coli* [50]. For flavonoid production, *S. albidoflavus* spores were quantified, and an inoculum of 106 spores/mL was performed in triplicate in shake flasks with 25 mL of NL333 medium [51] and incubated for 120 h at 30 °C and 250 rpm.

### 4.2. Reagents and Biochemicals

All solvents used for solid-phase extraction and HPLC-DAD analysis were LC-MS grade from either Sigma-Aldrich (Madrid, Spain) or VWR Chemicals (Barcelona, Spain). Luteolin, diosmetin, chrysoeriol, eriodictyol, hesperetin, homoeriodictyol, and homohesperetin were provided by Extrasynthese (Genay, France).

### 4.3. Genes and Enzymes

Restriction enzymes and T4 DNA ligase were purchased from Thermo Fisher Scientific (Madrid, Spain). Synthetic genes for the following ORFs were synthesized by Explora Biotech (Venezia, Italy), after codon optimization: a gene encoding a hypothetical class I SAM-dependent *O*-methyltransferase (M444_29925) from *Streptomyces* sp. Mg1 (Genbank accession no. OR820610); a gene encoding a 3-deoxy-7-phosphoheptulonate synthase (dahp) from *Amycolatopsis balhimycina* (Genbank accession no. OR820611); *At4g26220* from *Arabidopsis thaliana* (Genbank accession no. OR820609). Other genes used in this study were WP_013066811 (for *TAL*), NP_628552 (for *4CL*), L07647.1 (*CHS*), AY595413.1 (*CHI*), AY230247.1 (*FNS*), OQ674225 (*3′FH/CPR*).

### 4.4. Plasmids Construction

All the plasmids used in this study are listed in Table 1. Final constructs are depicted in Figure S5.

#### 4.4.1. Construction of pSEVAUO-M21503-FNS1/F3′H-CPR

The gene encoding FNS1 was assembled in a level 1 MoClo reaction using the level 0 plasmids PCR-Blunt II-TOPO-FNS1 [24] pSEVA181SP43, pSEVA181RiboJ-RBS, pIDTSMARTttsbib [21], and the level 1 MoClo receptor pSEVAUO-M21102 [21], yielding the plasmid pSEVAUO- M21102–FNS1. Then, this plasmid was used in combination with the plasmid pSEVAUO-M21206F3H-CPR and the level 2 MoClo receptor pSEVAUO-M21503 [21] to generate the plasmid pSEVAUO-M21503-FNS1/F3′H-CPR.

#### 4.4.2. Construction of pSEVAUO-M31105-At4g26220, pSEVAUO-M31105-M444_29925, and pSEVAUO-M31205-dahp

pSEVAUO-M31105-At4g26220 and pSEVAUO-M31105-M444_29925 are level 1 MoClo plasmids, and they were assembled by combining the level 1 MoClo receptor pSEVAUO-M31105 and the level 0 plasmids pSEVA181SP25, pSEVA181RiboJ-RBS, pIDTSMARTttsbib [21], and pSEVA181-At4g26220 (this study) or pSEVA181-M444_29925 (this study), respectively.

The plasmid pSEVAUO-M31205-dahp was assembled using the level 1 MoClo receptor pSEVAUO-M31205 and the level 0 plasmids pSEVA181SP25, pSEVA181RiboJ-RBS, pIDTSMARTttsbib, and pSEVA181-dahp (this study).

#### 4.4.3. Construction of pSEVAUO-M31505-At4g26220-dahp and pSEVAUO-M31505-M444_29925-dahp

The *At4g26220* and *M444_29925* genes were brought together with the *dahp* gene in two level 2 MoClo reactions. The first reaction was performed with the plasmids pSEVAUO-M31105-At4g26220, pSEVAUO-M31205-dahp, and the level 2 MoClo receptor pSEVAUO-M31505 [21], resulting in pSEVAUO-M31505-At4g26220-dahp. The second reaction was performed using the plasmids pSEVAUO-M31105-M444_29925 and pSEVAUO-M31205-dahp, yielding the plasmid pSEVAUO-M31505-M444_29925-dahp.

### 4.5. Flavonoid Extraction and LC-DAD Analysis

Spores from the different *S. albidoflavus* strains were incubated, as described before. Flavonoids were recuperated by organic extraction with acetone (cellular pellet) and ethyl acetate (culture supernatant). A sample of 1 mL was taken from the flasks and centrifuged at 12,000 rpm for 1 min to separate the supernatant from the pellet. The pellet was extracted with 1 mL of acetone using vortex for 1 h. The supernatant was extracted with 800 µL of ethyl acetate by agitation for 10 min. Both pellet and supernatant extractions were centrifuged for 1 min at 12,000 rpm, and the organic fractions were mixed and dried in a speed-vac. A second extraction was performed using 800 µL ethyl acetate over the cellular pellet and the supernatant using vortex and agitation, respectively, as described before. Finally, these extractions (cellular pellet and supernatant) were mixed with the initial dry extract obtained in the first extraction and dried in a speed-vac.

For the identification of flavonoids using HPLC-DAD, the final dry extract obtained from each cultivation condition was dissolved in 100 µL DMSO/MeOH 1:1 (*v*/*v*), and the samples were centrifuged prior to injection into the equipment. The HPLC separation was performed on an HPLC (1260 Infinity, Agilent Technologies, Madrid, Spain) equipped with an analytical column Pursuit XRs C18 (50 × 4.0 mm, 5 μm, Agilent Technologies, Madrid, Spain). HPLC gradient was made with analytical grade solvent B (acetonitrile (VWR, Barcelona, Spain), and water as solvents (1 mL/min flow rate). All solvents contained 0.1% formic acid. Samples were run by an isocratic elution of 10% MeCN from 0 min to 5.44 min, followed by a linear gradient from 10% to 35% of MeCN from min 5.44 to min 21.77, maintaining the mobile phase composition until 27.21 min. Then, a linear gradient from 35% to 100% MeCN between 27.21 min and 43.54 min was applied, followed by an isocratic elution until 55 min. Then, a linear gradient from 100% to 10% MeCN was applied from 55 min to 56 min. Finally, this mobile phase composition was maintained until the end of the program (61). Detection and spectral characterization of peaks were carried out with a photodiode array detector, and the analysis was performed with Data Analysis 4.3 software (Bruker, Billerica, MA, USA). All chromatograms were extracted at 280 nm. The column temperature was set to 30 °C. Flavonoids luteolin, diosmetin, chrysoeriol, hesperetin, homoeriodictyol, and homohesperetin were identified using authentic commercial standards. Luteolin, diosmetin, chrysoeriol, and hesperetin were quantified by comparing the peak area with that of a known amount of an authentic compound through a calibration curve. The production titers are expressed in mg/L, and the mean value was calculated from three biological replicates.

### 4.6. Statistical Analysis

Two-way ANOVA (analysis of variance) with Sidak’s multiple comparisons test was used to test the differences in the biosynthesis of diosmetin among the strains *S. albidoflavus* UO-FLAV-004-DIO and *S. albidoflavus* UO-FLAV-004-DIO-dahp and the biosynthesis of chrysoeriol among the strain *S. albidoflavus* UO-FLAV-004-CHR and the strain *S. albidoflavus* UO-FLAV-004-CHR-dahp. Graphical representation of the different generated data was carried out using GraphPad Prism software (version 9.0.2, GraphPad Software, San Diego, CA, USA), with a *p*-value < 0.05 considered as statistically significant (* *p* < 0.05; ** *p* < 0.005; *** *p* < 0.0005; **** *p* < 0.0001).

## Data Availability

Data and materials can be obtained from the research group upon request. Sequences accession numbers have been included in the Section 4.

## Supplementary Materials

The following supporting information can be downloaded at: https://www.mdpi.com/article/10.3390/ijms25052776/s1.
