# Supplementary figures and images for: Use of 3-Deoxy-D-arabino-heptulosonic acid 7-phosphate Synthase (DAHP Synthase) to Enhance the Heterologous Biosynthesis of Diosmetin and Chrysoeriol in an Engineered Strain of Streptomyces albidoflavus
Source: Int J Mol Sci. 2024 Feb 28;25(5):2776. doi: 10.3390/ijms25052776 (PMC10931780; doi:10.3390/ijms25052776)

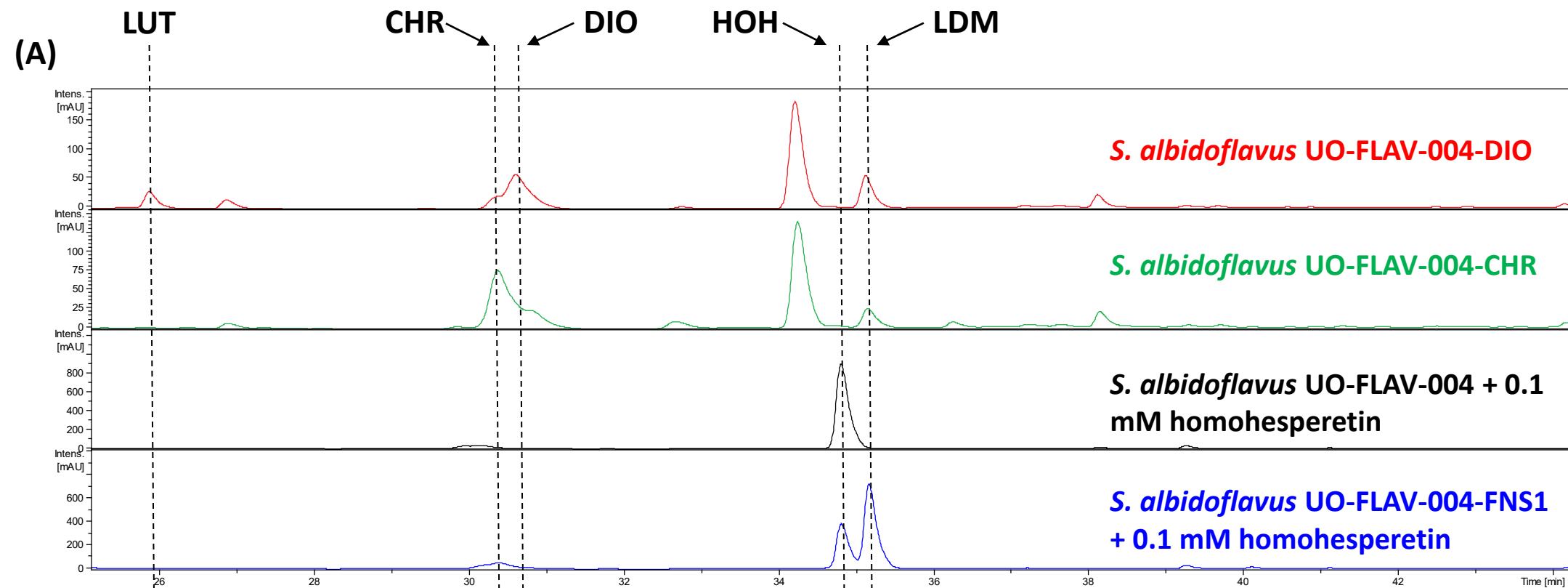

**(B)**

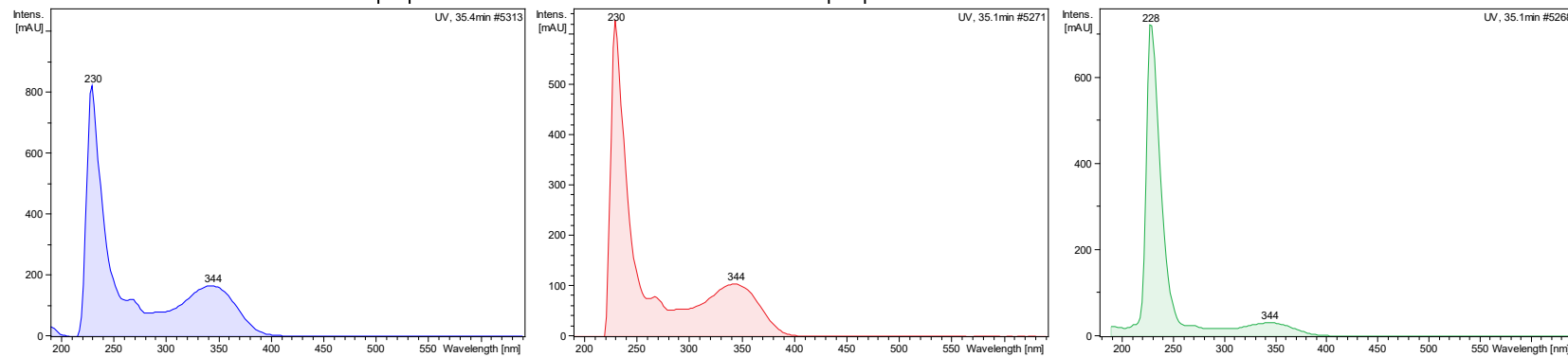

Supplement: Supplementary file 1 [file ijms-25-02776-s001.zip › Figure S1.pdf]

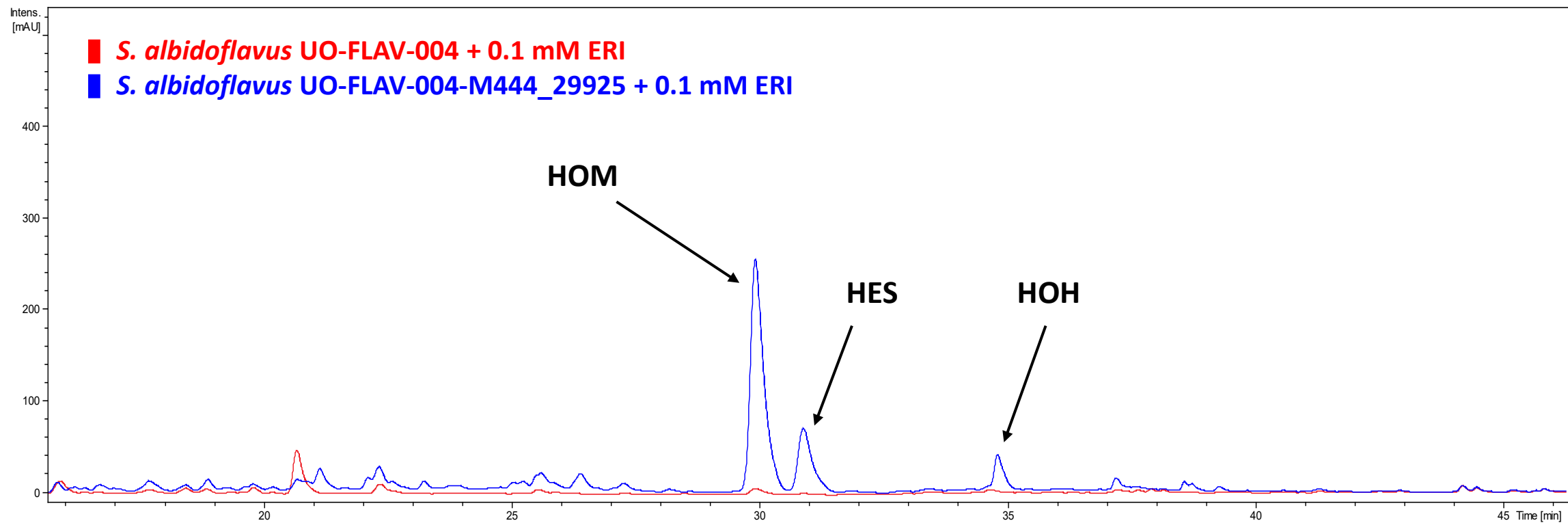

Supplement: Supplementary file 1 [file ijms-25-02776-s001.zip › Figure S2.pdf]

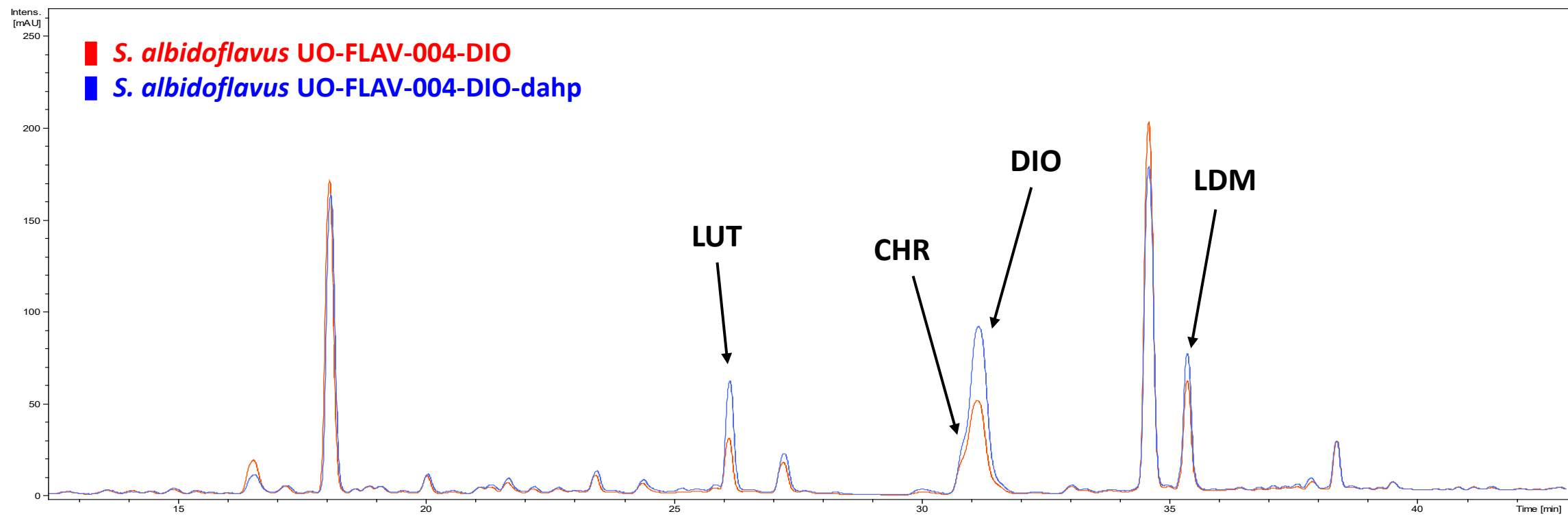

Supplement: Supplementary file 1 [file ijms-25-02776-s001.zip › Figure S3.pdf]

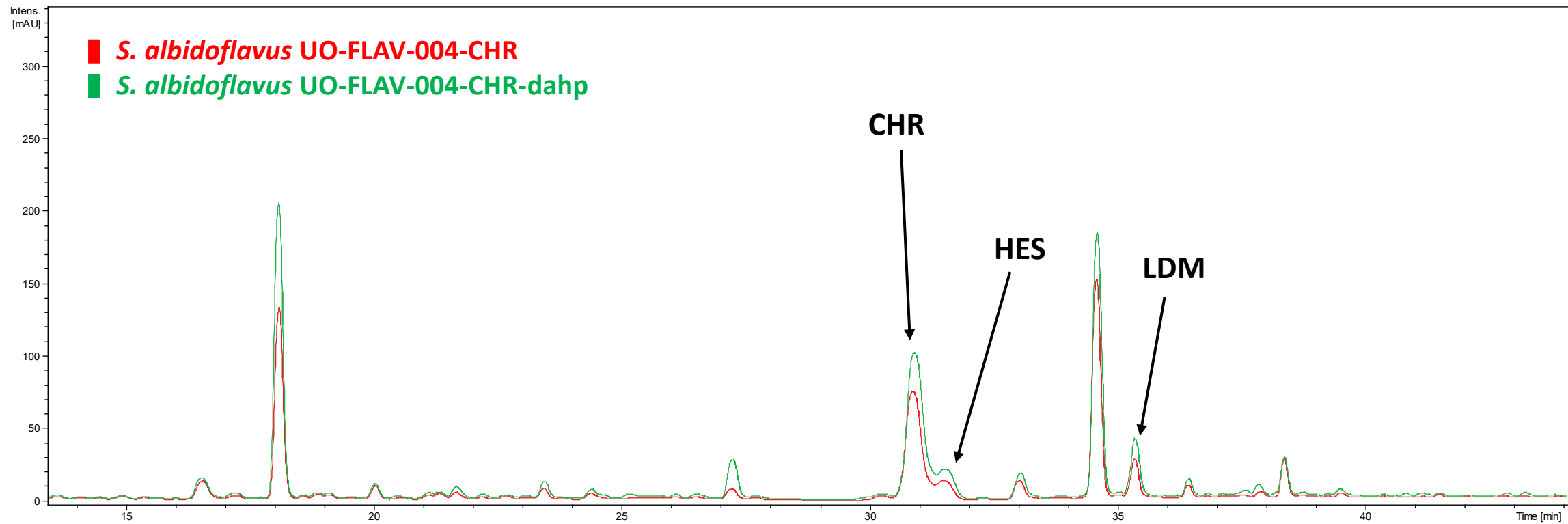

Supplement: Supplementary file 1 [file ijms-25-02776-s001.zip › Figure S4.pdf]
